# Optimal treatment of multivessel complex coronary artery disease

**DOI:** 10.3892/etm.2014.1630

**Published:** 2014-03-21

**Authors:** HAIHUI SUN, LIANQUN CUI

**Affiliations:** 1 Department of Cardiology, The Provincial Hospital Affiliated to Shandong University, Jinan, Shandong 250021, P.R. China; 2 Department of Cardiology, The Central Hospital of Tai’an, Tai’an, Shandong 271000, P.R. China

**Keywords:** multivessel coronary artery disease, complete revascularization, incomplete revascularization, prognosis

## Abstract

The aim of the present study was to investigate major cardiac events and the similarities and differences of medical costs among patients with multivessel complex coronary artery disease (MCCAD) during the three-year follow-up. The MCCAD patients had undergone single complete revascularization (CR), fractionated revascularization (FR) or partial revascularization (PR) and the present study aimed to screen the optimal treatment program. A total of 2,309 MCCAD patients who had been treated at a single center in the last decade, among which 1,020 cases underwent single CR, 856 cases successively underwent FR and 433 cases only underwent PR, were followed-up for three years. Major cardiac events, including all-cause mortality, myocardial infarction, severe heart failure, rehospitalization and revascularization (coronary artery bypass grafting and coronary stent reimplantation), were set as the end points. In addition, the three-year medical costs associated with heart disease were analyzed. The three-year cardiac event rate in the CR group (17%) was significantly lower compared with the other two groups and the average three-year medical costs in the CR group (62,100 RMB) were significantly lower than those in the other two groups. Therefore, under permissive conditions, single CR is the optimal and most economical treatment strategy for patients with MCCAD.

## Introduction

With the increasing incidence of coronary artery disease (CAD), coronary angiography, as the gold standard for CAD diagnosis, has obtained growing popularity. An increasing number of hospitals have acquired the ability and qualifications to perform coronary angiography. Through clinical studies, it has been identified that multivessel lesions are common in stable angina and acute coronary syndrome (1,2), and are an independent predictor of CAD that affects the prognosis of patients (3). Controversy remains with regard to the treatment strategies for patients with multivessel complex coronary artery disease (MCCAD), particularly in recent years. Since the one-step implantation of multi-stents has been charged and limited, it remains inconclusive whether patients with MCCAD should undergo single complete revascularization (CR), fractionated revascularization (FR) or partial revascularization (PR). The present retrospective study compared the effects of the various revascularization modes (complete, fractionated or partial) with regard to the long-term prognosis of patients with MCCAD. Similarities and differences in the medical costs were compared with the aim of screening the optimal treatment program for patients with MCCAD.

## Materials and methods

### Study population

A total of 2,309 patients with CAD that had been admitted to the Department of Cardiology at the Provincial Hospital Affiliated to Shandong University (Jinan, China) between December 2003 and October 2009 were selected for the study. The patients were aged between 41 and 78 years with a mean age of 59±10 years. Patients were divided into the CR (1,020 cases), FR (856 cases) and PR groups (433 cases). The three groups all underwent selective coronary angiography and percutaneous coronary intervention (PCI) surgery due to the severe coronary artery stenosis; each patient was implanted with at least one stent. Exclusion criteria included acute ST-segment elevation-induced myocardial infarction, valvular heart disease accompanied with heart failure and congenital heart disease accompanied with clearly diagnosed cancer. In addition, if the patient or their families refused the stent implantation, or were unable to be followed-up for various reasons, these patients were excluded from the study. Written informed consent was obtained from the patient’s family.

### Assessment of coronary angiography and stent implantation

Surgical procedures were performed strictly in accordance with the American Heart Association/American Heart Association Coronary Angiography Guidelines. A Philips cardiovascular imaging machine (Koninklijke Philips N.V., Amsterdam, Netherlands) and Siemens cardiovascular imaging machine (Siemens AG, Munich, Germany) were used for selective coronary angiography via puncture of the right femoral artery or right radial artery. The surgical procedures were conducted strictly in accordance with the standard interventional procedures (4). CAD diagnosis was determined based on the results of the coronary angiography, namely, ≥50% stenosis of at least one major coronary artery. MCCAD included unprotected left main coronary artery disease, multivessel disease (MVD), chronic total occlusion disease (CTO), diffuse long lesion and bifurcated disease, among which MVD was defined as at least two main branches of the epicardial coronary artery or the major branches with ≥50% stenotic lesions (5). Diffuse long lesion was defined as a lesion with >20 mm single length. CTO was diagnosed if the coronary artery was completely occlusive and lasted for more than three months. Successful intracoronary stenting criteria were as follows: <20% post-stenting residual stenosis of the target vessel lumen and Thrombolysis In Myocardial Infarction Grade Flow III. CR referred to <50% post-PCI residual stenosis of all the major coronary arteries and their branches (6), while ≥50% residual stenosis of any coronary artery and its branches was defined as PR. FR referred to the achievement of CR of the target coronary vessel via a FR procedure.

### Drug therapy

All patients underwent preoperative drug therapy with aspirin (100 mg/day) and clopidogrel (75 mg/day). Clopidogrel (300–600 mg loading dose) was administered one day prior to surgery to all patients excluding those who had obtained long-term antiplatelet therapy outside the Provincial Hospital Affiliated to Shandong University. Postoperative clopidogrel (75 mg/day) was administered for at least one year and long-term administration of aspirin (100 mg/day) and blood fat-regulating drugs, including statin-category drugs, was recommended.

### Follow-up and end-point determination

The follow-up time was 36 months maximum and was conducted via regular clinics, telephone contact or recoronary angiography. The occurrence of angina pectoris and major adverse cardiac events was recorded, including nonfatal myocardial infarction, heart failure and mortality (cardiac or non-cardiac), rehospitalization due to the aforementioned reasons and revascularization by coronary artery bypass grafting or recoronary artery stenting. End-point determination primarily relied on inquiring with the patients or their families, consulting the medical admission doctors, evaluating medical records and associated auxiliary examinations and laboratorially examining the indexes. The primary end point was all-cause mortality within three years, while secondary end points were complex end-point events, including nonfatal myocardial infarction, heart failure, rehospitalization due to the aforementioned reasons and revascularization (coronary artery bypass grafting and recoronary artery stenting). All-cause mortality included cardiac and non-cardiac mortality. Myocardial infarction included ST-segment elevation-induced and non-ST-segment elevation-induced myocardial infarction and was defined as an increase or decrease in the levels of cardiac biomarkers (preferably cardiac troponin) by >99% of the upper reference limit of 0.09 ng/ml. It was also accompanied by at least one of the following symptoms of myocardial ischemia: Ischemic symptoms, electrocardiography (ECG)-prompted new ischemic changes, ECG-prompted pathological Q wave or radiographic evidence indicating new regional wall motion abnormalities or the loss of viable myocardium. Revascularization was vascularization performed more than three months after the first vascularization, including target and non-target vessel-revascularization.

### Statistical methods

SPSS 13.0 software (SPSS, Inc., Chicago, IL, USA) was used for statistical analysis. Measurement data are expressed as the mean ± SD and were analyzed with the t-test. The χ^2^ test was used to analyze the counting data. Logistic regression multivariate correlation analysis was also performed and P<0.05 was considered to indicate a statistically significant difference. The survival curves were estimated with Kaplan-Meier.

## Results

### Comparison of clinical features

No significant differences were identified with regard to the gender ratio, hypertension, hypercholesterolemia and smoking habits among the three groups (P>0.05). However, the average age, prevalence of diabetes and incidence of remote myocardial infarction and left ventricular dysfunction in the PR and FR groups were higher than those in the CR group and the differences were statistically significant (P<0.05; Table I).

### Comparison of CAD, stent implantation and medical costs

Compared with the CR group, the mean number of lesions, average lesion stenosis and the number of patients with severe stenosis, complex lesions, three-branch lesions, left main stem disease, bifurcated lesions and CTO in the PR and FR groups were significantly higher (P<0.05), while the average number of stents was significantly lower (P<0.05). No significant differences were observed in the proportion of long lesions and the average length of the stents among the groups (P>0.05; Table II).

### Comparison of follow-up observations

**Figure 1:**
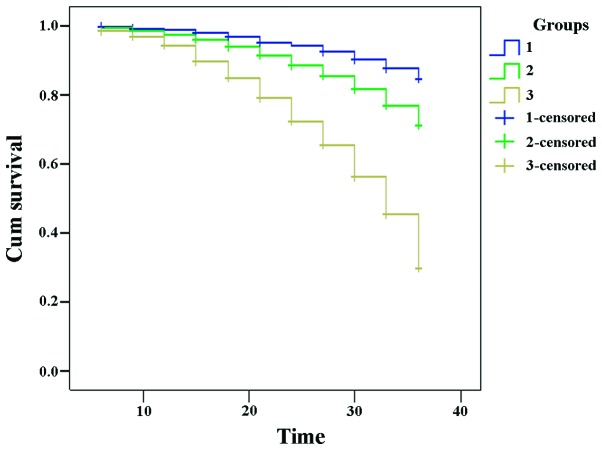
Survival curves of the patient in the CR, PR and FR groups. Group 1, CR; 2, FR; and 3, PR. Cum, cumulative; CR, complete revascularization; FR, fractionated revascularization; PR, partial revascularization.

The follow-up period was 36 months. During the follow-up period, no significant difference was observed in the in-stent restenosis rate among the three groups (P>0.05), while statistically significant differences were identified in the number of cases of recurrent angina, myocardial infarction, heart failure, revascularization and all-cause mortality among the three groups (P<0.05; Table III). The incidence of major adverse cardiac events during the three-year follow-up period in the CR group (17%) was significantly lower than that in the FR (29%; P<0.01) and PR groups (67%; P<0.001). In addition, the three-year survival rate in the CR group was significantly longer than the rates in the FR and PR groups and the difference among the survival times was statistically significant (P<0.001; Table IV; Fig. 1). The three-year medical costs in the CR group (62,100 RMB) were significantly lower than those in the FR (83,200 RMB; P<0.001) and PR groups (66,900 RMB; P<0.01).

### Analysis of the survival rates

Survival times of the three groups were statistically compared (χ^2^ = 487.968; P<0.001) and statistically significant differences were observed. The survival rate is the highest in CR, and the lowest in partial revascularization, and the middle is FR.

## Discussion

The incidence of CAD has gradually increased and the age of onset has become increasingly younger. MCCAD is one of the most serious types of CAD that commonly leads to complications, including heart enlargement, heart failure, malignant arrhythmias and cardiac sudden death, which seriously impacts the quality of life and life expectancy of a patient. The treatment principles of CAD include drug therapy, reperfusion therapy and heart transplantation. Drug therapy is the basis in the treatment of CAD. Based on positive drug intervention, therapy which is able to timely open the coronary blood vessels and ensure continuous and effective levels of myocardial reperfusion may significantly reduce the myocardial ischemic area, rescue heart function, reduce mortality and complications and improve the prognosis of patients. PCI treatment has become the most important method of myocardial revascularization. Patients with MCCAD constitute the high-risk population for serious cardiovascular events and target vessel revascularization. Myocardial blood supply should be actively improved, preventing left ventricular remodeling, protecting the function of the heart, reducing major cardiac events, including arrhythmia, heart failure and sudden mortality, and CR should be achieved to the greatest extent possible. Controversy remains with regard to achieving CR in patients with MCCAD (7,8). The risks of postoperative elevated serum creatine kinase levels, contrast-induced nephropathy and thrombosis are likely to increase in MCCAD patients, compared with other patients (9–11). An American three-year follow-up study involving >20,000 individuals identified that the risk of mortality following PR was significantly increased when compared with CR (12). The majority of foreign studies have demonstrated that for patients with MCCAD, regardless of the surgical bypass or medical intervention therapy administered, achieving CR to the greatest possible extent significantly improves prognosis (13,14). As for MCCAD patients undergoing a coronary artery bypass graft, CR was the most successful vascular reopen strategy (15–18). In the present study, retrospective analysis was performed to compare the prognoses of three treatment groups. The results revealed that throughout the follow-up period, the rates of recurrent angina, nonfatal myocardial infarction, heart failure, rehospitalization due to the aforementioned reasons and revascularization (coronary artery bypass grafting and coronary stent reimplantation) in the PR group were statistically significantly higher than those in the CR group, indicating that the short and long-term prognoses of CR were better compared with those of PR. These results are consistent with the conclusions of the majority of foreign studies (19–21). The three-year medical costs of the CR group were significantly lower than those of the PR and FR groups. The complexity of CAD, degree of stenosis, previous history of revascularization and cases of remote myocardial infarction in the PR group were higher compared with those in the CR group (P<0.05). These observations indicated that the heart conditions of the PR patients were worse than those of the CR patients, the risk factors were increased, the left ventricular ejection fraction was reduced and CR was unable to be achieved due to the disease condition and technical reasons. With the current improvements to PCI surgical technology, an increasing number of patients with MCCAD may achieve CR. Therefore, the difficulty of performing PCI in PR patients is markedly higher than in CR patients, which may also be one of the factors causing the difference in the long-term prognoses. Furthermore, during PCI, the present study identified that CR was difficult to achieve in certain coronary arteries due to the following reasons: Diffuse vascular disease, vascular calcification, distal lesions or small branch lesions; CTO lesions with long history while the guiding wire, balloon or stent could not pass; the vessels were seriously distorted or calcified so that the guiding wire, balloon or stent could not pass, or the stent expansion was poor, and was not able to adhere to the walls; and partial left main lesions. Tolerance to the surgery and economic conditions of the patient were also factors resulting in a PR outcome. In clinical practice, each case should be carefully analyzed, the appropriate surgical instruments selected, the surgical techniques and methods improved and the positive and negative points fully balanced, thereby improving the success rate of complex-lesion PCI surgery and reducing complications. In conclusion, the present study demonstrated that the long-term prognosis of CR implementation through PCI for patients with MCCAD was better compared with that of PR.

## Figures and Tables

**Table I:** Comparison of the clinical data of among three groups.

Group	Cases, n	Age, years	Males, n (%)	Smoker, n (%)	Hypertension, n (%)	Hypercholesterolemia, n (%)	Diabetes, n (%)	CAD family history, n (%)	Left ventricle ejection ratio, %	Remote myocardial infarction, n (%)	Unstable angina, n (%)
CR	1020	58±10	753 (73.8)	247 (24.2)	676 (66.3)	623 (61.1)	268 (26.3)	196 (19.3)	64±11	180 (17.6)	744 (73.0)
FR	856	61±9	615 (71.9)	158 (18.5)	525 (61.3)	504 (58.9)	320 (37.4)	218 (25.5)	53±10	218 (25.5)	590 (68.9)
PR	433	62±9	304 (70.4)	84 (19.4)	275 (63.5)	252 (58.2)	174 (40.2)	105 (24.2)	52±11	125 (28.9)	294 (67.9)

CAD, coronary artery disease; CR, complete revascularization; FR, fractionated revascularization; PR, partial revascularization.

**Table II:** Comparison of coronary lesions, stenting indicators and medical costs.

Group	Cases, n	Lesions, n	Average lesion stenosis, %	Severe stenosis lesion, n (%)	Complex lesion, n (%)	Three-branch lesion, n (%)	Left main stem lesion, n (%)	Bifurcated lesion, n (%)	CTO, n (%)	Long lesion, n (%)	Stents, n	Stent length, mm
CR	1020	2.6±0.9	75±11	432 (42.4)	655 (64.2)	295 (28.9)	25 (2.5)	153 (15.0)	105 (10.3)	236 (23.1)	2.5±1.5	18.8±5.6
FR	856	3.3±1.2	84±10	443 (51.8)	681 (79.5)	456 (53.2)	77 (8.9)	188 (21.9)	205 (23.9)	188 (21.9)	1.4±0.8	19.1±6.2
PR	433	3.4±1.2	85±10	226 (52.2)	344 (79.4)	235 (54.2)	42 (9.7)	95 (21.9)	108 (24.9)	103 (23.8)	1.9±0.9	18.9±5.7

CTO, chronic total occlusion disease; CR, complete revascularization; FR, fractionated revascularization; PR, partial revascularization.

**Table III:** Comparison of follow-up results.

Group	Cases, n	In-stent restenosis, n (%)	Angina, n (%)	Myocardial infarction, n (%)	Heart failure, n (%)	Revascularization (unchanged number), n (%)	All-cause mortality, n (%)	MACE events, n (%)	Medical costs, RMB
CR	1020	121 (11.9)	307 (30.1)	18 (1.7)	67 (6.5)	142 (13.9)	12 (1.2)	173 (17)	62,100
FR	856	111 (12.9)	246 (28.7)	19 (2.2)	84 (9.8)	111 (12.9)	15 (1.8)	248 (29)	83,200
PR	433	63 (14.6)	191 (44.1)	12 (2.8)	93 (21.4)	98 (22.8)	17 (3.9)	290 (67)	66,900

MACE, major adverse cardiac events; CR, complete revascularization; FR, fractionated revascularization; PR, partial revascularization.

**Table IV:** Statistical comparison of the survival times among the three groups.

Group	Mean survival time (months)	RSD
CR	34.544	0.144
FR	33.255	0.212
PR	29.449	0.397
Overall	33.153	0.130

RSD, relative standard deviation; CR, complete revascularization; FR, fractionated revascularization; PR, partial revascularization.
